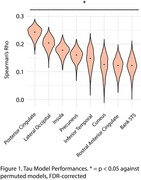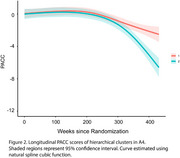# Whole‐brain functional connectivity predicts regional tau PET and future cognitive decline in preclinical Alzheimer's disease

**DOI:** 10.1002/alz70856_100150

**Published:** 2025-12-25

**Authors:** Hamid Abuwarda

**Affiliations:** ^1^ Yale School of Medicine, New Haven, CT, USA

## Abstract

**Background:**

Preclinical Alzheimer's disease (AD) is characterized by abnormal amyloid accumulation without cognitive symptoms. While individuals with preclinical AD show functional connectivity changes, it is unclear whether functional connectivity is a predictor of AD pathology. We used connectome‐based predictive modeling (CPM) using functional connectivity to predict focal tau deposition and identify clinically meaningful subgroups based in a preclinical AD cohort.

**Method:**

We used CPM to predict baseline tau (18F‐flortaucipir) and amyloid PET (18F‐florbetapir) from the resting‐state functional connectome in the Anti‐Amyloid in Asymptomatic Alzheimer's disease study (A4, *n* = 394, aged 65‐85). A two‐component gaussian mixture model was used to separate tau positive binding from non‐specific signal. CPM performances were assessed using Spearman's ⍴ of predicted tau binding vs. observed values. Significance was assessed against permuted models (*n* = 1000 iterations) and FDR‐corrected. Models were validated in the Alzheimer's Disease Neuroimaging Initiative (ADNI, *n* = 469, aged 55‐90). Using edges from the most predictive models (⍴ >0.20), we performed hierarchical clustering in a separate cohort of individuals from the A4 placebo arm who were not included in the original models. The analysis controlled for normal age‐ and sex‐related connectivity changes. We compared longitudinal PACC scores between clusters using a linear mixed‐effects (LME) model.

**Result:**

Whole‐brain functional connectivity robustly predicted regional tau PET, outperforming amyloid PET models. The best‐performing tau models (posterior cingulate ⍴=0.24, lateral occipital ⍴ = 0.20; insula ⍴ = 0.18, *p* <0.05 against permuted models) generalized to ADNI, specifically in participants who had elevated levels of tau (SUVR >1.19). Hierarchical clustering of the predictive edges from posterior cingulate and lateral occipital models revealed 2 distinct clusters in the A4 dataset. LME analysis showed that one cluster had a significantly faster decline (group * time interaction, *p* <0.0001).

**Conclusion:**

The functional connectome modestly predicts regional tau PET across the AD spectrum. These predictive edges capture meaningful differences in cognitive trajectories, underscoring the relationship between functional connectomic changes, tau burden, and clinical trajectory. Robust prediction of regional tau that correlate with cognitive trajectories suggest that functional connectivity is valuable for early disease staging. Future studies should investigate whether functional connectivity can be combined with other biomarkers to enhance staging accuracy.